# Multisystemic Impact of RNF213 Arg4810Lys: A Comprehensive Review of Moyamoya Disease and Associated Vasculopathies

**DOI:** 10.3390/ijms26167864

**Published:** 2025-08-14

**Authors:** Eva Bagyinszky, YoungSoon Yang, Seong Soo A. An

**Affiliations:** 1 Department of Industrial and Environmental Engineering, Graduate School of Environment, Gachon University, Seongnam 13120, Republic of Korea; eva85@gachon.ac.kr; 2 Department of Neurology, Soonchunhyang University Hospital, Cheonan 31151, Republic of Korea; 3 Department of Bionano Technology, Gachon Medical Research Institute, Gachon University, Mirae 1 Building, 1342 Seongnamdaero, Sujeong-gu, Seongnam 13210, Republic of Korea

**Keywords:** RNF213, RNF123 Arg4810Lys, Moyamoya disease, vascular diseases, risk modifiers

## Abstract

The ring finger protein 213 (RNF213) Arg4810Lys variant has been previously identified as a significant risk factor for Moyamoya disease (MMD), particularly in East Asian populations. This review explores the broader impact of the Arg4810Lys mutation on various cerebrovascular conditions, including Moyamoya syndrome (MMS), intracranial artery stenosis, quasi-Moyamoya syndromes, ischemic stroke, and intracranial atherosclerosis. Beyond the brain, it is also implicated in pulmonary arterial hypertension, coronary artery disease, and renal artery stenosis, emphasizing its systemic effects. Functional studies suggest that RNF213 Arg4810Lys alters angiogenic signaling, endothelial cell function, vascular remodeling, and immune response pathways, especially when influenced by environmental stressors, like hypoxia or inflammation. The gene dosage of Arg4810Lys significantly affects disease phenotypes, with homozygous carriers typically experiencing earlier onset with increased severe symptoms. The variant also exhibits incomplete penetrance and frequently co-occurs with additional genetic alterations, including trisomy, KIF1A, FLNA, and PCSK9 mutations, which complicates its pathogenicity. A comprehensive understanding of RNF213 Arg4810Lys’s systemic impact is essential to developing effective risk assessment strategies, personalized treatments, and targeted therapies for associated vascular diseases.

## 1. Introduction

Moyamoya disease (MMD) is a progressive steno-occlusive cerebrovascular disorder that can lead to vascular cognitive impairment (VCI). This impairment is primarily caused by chronic cerebral hypoperfusion and recurrent microinfarctions. The term “Moyamoya” itself is Japanese for “hazy” or “a puff of smoke,” aptly describing the characteristic angiographic appearance. MMD is characterized by the progressive narrowing or occlusion of the terminal portions of the internal carotid arteries (ICAs) and the proximal segments of the middle cerebral arteries (MCAs). This progressive stenosis produces the distinctive “puff of smoke” appearance due to the formation of a compensatory collateral network of fragile vessels [1,2,3,4,5,6,7,8,9,10,11,12,13,14].

The narrowing of arteries inside the skull due to fatty deposits, particularly in the ICAs, is a serious condition associated with depression, cognitive dysfunction, and seizures. Consequently, reductions in cerebral blood flow lead to chronic hypoxia and metabolic insufficiency in the brain, resulting in gradual neuronal injury. This hypoperfusion predominantly affects the frontal lobes (impairing executive functions), temporal lobes (affecting memory), and parietal lobes (impacting visuospatial processing), thereby contributing to progressive cognitive decline. These steno-occlusive changes are associated with a high risk of transient ischemic attacks (TIAs), cerebral infarction, or intracranial hemorrhage [14,15].

### 1.1. Cognitive Manifestations of MMD

Memory dysfunction in MMD is primarily characterized by impaired working memory and short-term recall. Unlike the amnestic profile of Alzheimer’s disease (AD), where new information is difficult to store, MMD patients often struggle with retrieving already-stored information. This is linked with chronic hypoperfusion or recurrent ischemic insults in the temporal lobes. MMD can appear as a bilateral condition, and when both cerebral hemispheres are affected, cognitive impairment may be more pronounced and may progress more rapidly [16,17,18,19,20,21,22,23,24,25,26,27]. Executive dysfunction is one of the most prominent cognitive domains affected in MMD. Patients frequently experience a decline in abilities like planning, problem solving, attention, and task switching, all of which are related to frontal lobe hypoperfusion. These deficits often lead to difficulties with daily functioning, coping with new situations, and a decrease in judgment and situational awareness [16,17,18,19,20,21,22,23,24,25,26,27]. MMD can also lead to a gradually progressing form of vascular cognitive impairment, which presents as a subcortical vascular dementia phenotype distinct from typical AD. The clinical presentation is often multifactorial and evolves over time. Early identification through comprehensive neuropsychological testing and advanced neuroimaging, such as MRI, perfusion SPECT, and PET, is essential to accurate diagnosis and management [16,21].

Additionally, a decrease in cognitive processing speed is a prominent feature, which is attributable to white matter injury, secondary to chronic hypoperfusion or small-vessel ischemia. Patients often present with slowed verbal responses, reduced psychomotor speed, and general mental sluggishness. Deficits in attention and concentration, further compromising memory encoding and task performance, are frequently reported. Patients have difficulties in maintaining their attention, are easily distractible, and demonstrate reduced cognitive efficiency in both structured and unstructured environments [16,17,23,24]. Neuropsychiatric symptoms, such as apathy, depressive features, and emotional blunting, are also common. These are likely secondary to the disruption of fronto-subcortical circuits and are characterized by diminished affective responsiveness, decreased motivation, and reduced engagement in goal-directed activities [17,18].

### 1.2. Epidemiology and Genetics of MMD

MMD has become a major causative factor for ischemic stroke among Asian, African American, and Hispanic populations, but it is less common among Caucasians [10,11,12,13]. It is particularly prevalent among East Asians, especially Korean and Japanese populations [14].

**Figure 1:**
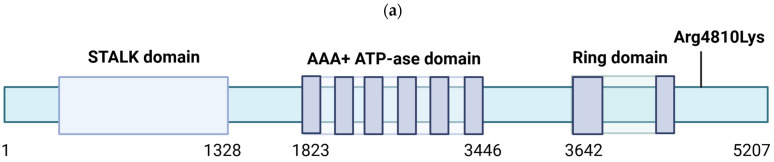

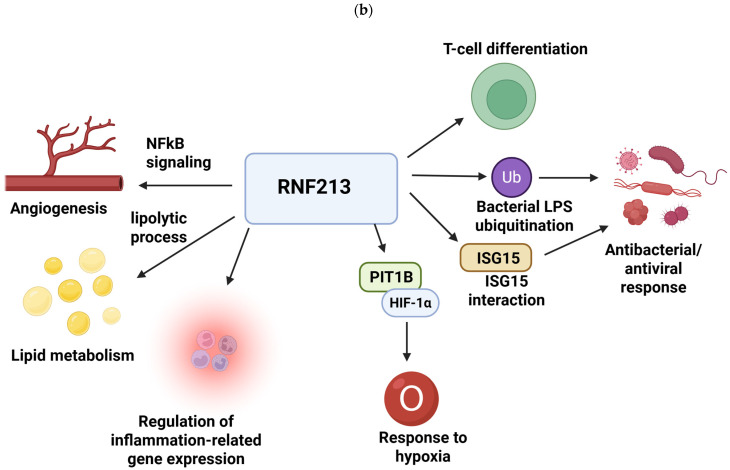
(**a**). Structure of *RNF213* gene and the location of the Arg4810 mutation. (**b**). Functions of the RNF213 gene. RNF213 has been found to be a multifunctional gene which may impact angiogenesis, lipid metabolism, inflammation regulation, cytoskeletal organization of smooth muscle cells, hypoxia response, and protection against microbes [35].

The ring finger protein 213 (*RNF213*) gene is implicated in MMD and several other vascular diseases, including intracranial artery stenosis (ICAS). Located on chromosome 17q25.3, the *RNF213* gene encodes a 591 kDa cytosolic protein. The RNF213 protein, also known as “mysterin”, contains two domains exhibiting ATPase and ubiquitin ligase activities [1,2]. Although its exact mechanisms remain unclear, RNF213 is implicated in the development of blood vessels [3,4]. RNF213 is a 5207-amino acid protein composed of three distinct regions: a long “arm” at the N-terminus, a ring-shaped domain in the middle containing the ATPase “motor,” and the E3 enzyme module at the C-terminus. Additionally, RNF213 has a dynein-like structure with six AAA domains within a single polypeptide chain (Figure 1a) [2,3,4].

The N-terminal “stalk” domain has been suggested to be involved in protecting against infections, including pathogen recognition, but may not participate in ubiquitination-related pathways. The AAA ring shares structural similarity with dynein; among these domains, AAA3 and AAA4 have been verified to bind and hydrolyze ATP and ubiquitination, while AAA2 has been suggested to contain an ATP-binding site without contributing to catalytic activity. The E3-RING domain, located at the top of the E3 scaffold, consists of E3-back, E3-shell, and E3-core subdomains. This domain functions as a ubiquitin ligase, catalyzing the ubiquitination of both proteins and lipids [2,3,5,6].

The RNF213 protein has been found to be widely expressed in the human body, especially in the immune cells or immune-related tissues (spleen or leukocytes), but RNF213 expression has also been found in cerebral endothelial cells, heart, placenta, or lymph nodes [14,28]. The RNF213 protein has been found to impact diverse pathways and could play a role in different pathways (Figure 1b). RNF212 has been suggested to impact the regulation of immune signals and could play a role in controlling the expression of inflammation-related genes. RNF123 has been found to be involved in angiogenesis through NFkappaB signaling. Knockdown of RNF213 can impair significantly the angiogenic process [6]. RNF213 has also been found to regulate lipid metabolism by suppressing the lipolytic process [6]. RNF213 has been found to be involved in the control of non-mitochondrial oxygen consumption and in the response for low oxygen levels (hypoxia) by interacting with different proteins, including Protein Tyrosine Phosphatase 1B (PTP1B) and Hypoxia Inducible Factor 1 Subunit Alpha (HIF1A) [29,30,31]. RNF213 also impacts the innate immune responses by acting as an interactor of interferon-stimulated gene 15 (ISG15) and a cellular sensor of ISGylated proteins. The interaction between RNF213 and ISG15 plays an important role in antiviral and antibacterial activities. This interaction is induced by interferon signals, including type I interferons (IFN-I) [5,7,8,9]. Furthermore, RNF213 has been found to act as a key immune sensor by catalyzing the ubiquitination of bacterial lipopolysaccharide (LPS), restricting the proliferation of cytosolic bacteria, including Salmonella. It can also promote regulatory T (Treg) cell differentiation, including peripheral natural killer (NK) cells and T cells, playing a crucial role in resisting various microbial infections [32,33]. Furthermore, RNF213 may control cytoskeletal organization and contractility in the vascular smooth muscle cells (vSMCs) [34].

### 1.3. Clinical Course and Management of MMD

The onset of MMD, whether in childhood or adulthood, is caused by reduced cerebral blood flow in various brain regions, including the basal ganglia. In childhood-onset MMD, symptoms often include stroke or recurrent TIAs, leading to weakness, motor impairment, paralysis, and difficulties with vision or language. Cerebral infarction is relatively rare in children with MMD [36]. In adult-onset MMD, cerebral hemorrhage is more common than ischemia; however, non-hemorrhagic forms, including cerebral ischemia (TIAs or infarctions), have also been reported, especially in patients younger than 30 years [34,37]. Currently, no cure exists for MMD, but surgical revascularization has been shown to improve symptoms [37,38].

Typically, the disease presents an autosomal dominant inheritance pattern, although homozygous forms of mutations have also been reported [14,39]. The *RNF213* Arg4810Lys variant (rs112735431) has been identified in multiple cases of vascular dysfunction, showing a strong association with MMD susceptibility. This mutation is commonly described in East Asian patients. Its inheritance reveals an autosomal dominant pattern but also exhibits incomplete penetrance [40,41,42,43,44]. The variant is located in the C-terminal region of RNF213, near the E3-RING domain, and likely affects its protein function [45]. Several studies have reported details of the clinical symptoms, phenotypes, and possible pathogenic mechanisms of the *RNF213* Arg4810Lys mutation. This review summarized the impact of this variant on vascular diseases, including MMD, and explored the potential pathogenic mechanisms involved.

## 2. *RNF213* Arg4810Lys and MMD: Biomarkers and Treatment Options

As mentioned above, the *RNF213* Arg4810Lys variant is a common and strong genetic risk factor for MMD [46,47,48,49,50]. Both homozygous and heterozygous forms have been found in MMD patients, but penetrance is higher for homozygous cases than heterozygous. Phenotypically, infarctions and transient ischemic attacks are more common in both homozygous and heterozygous Arg4810Lys carriers than in non-carriers. Posterior cerebral artery involvement is frequent in heterozygous carriers, while intracerebral/intraventricular hemorrhage is less common in heterozygotes than in non-carriers (Table 1). A family history of MMD is common among both heterozygous and homozygous carriers. A meta-analysis by Wang et al. (2021) suggested that *RNF213* Arg4810Lys could serve as a biomarker to classify different MMD phenotypes in both homozygous and heterozygous stages [51,52,53,54,55,56,57,58,59]. Jiang et al. (2023) [60] found that the Arg4810Lys variant was associated with younger disease onset, an increased risk of seizures, cerebral ischemia, and posterior cerebral artery involvement, but carriers showed a reduced risk of intracerebral/intraventricular hemorrhage [60]. In terms of vascular morphology, MMD patients with the Arg4810Lys variant exhibited a reduced carotid canal diameter compared with controls [61]. This variant is rare or absent in Caucasian, Southeast Asian, and other non-Asian populations [48,62,63,64,65,66,67], including its absence in Southeastern Asian populations [62].

### 2.1. Korean Studies on Arg4810Lys and MMD

Multiple Korean studies have reported on the Arg4810Lys mutation and its involvement in MMD. Jang et al. (2017) [68] screened 264 adult MMD cases with two control groups (622 and 1100 individuals), revealing that 67.4% of MMD patients had the Arg4810Lys mutation, significantly higher than in controls. However, no association was found between other rare *RNF213* variants and MMD in another study [68]. Given its high prevalence in Korean, Japanese, and Chinese MMD patients, *RNF213* Arg4810Lys is likely a founder mutation among East Asians [56,67,68,69,70,71].

In 2015, a Korean study of 165 MMD patients from 155 unrelated families reported a higher frequency of the Arg4810Lys variant at 75.5%, mostly heterozygous, with 10% homozygous cases. The mutation was more common in patients with a positive family history of MMD. Homozygous carriers had earlier disease onset (by at least 5 years), faster disease progression, more severe cognitive impairment, and a higher prevalence of epilepsy. This study suggested that the homozygous form could be a useful biomarker for early-onset or unstable MMD, requiring prompt diagnosis and therapeutic development [72]. Jee et al. (2020) reported that Arg4810Lys may be a risk factor for extracranial arteriopathy in young adults with probable MMD, especially in the homozygous form, independently of other vascular risk factors like diabetes mellitus [73]. Similarly, a 2023 Korean study observed that revascularization surgery was more common in homozygous carriers than in heterozygotes or non-carriers. Homozygous carriers presented with seizures more frequently, higher posterior cerebral artery involvement, and increased cerebral infarctions [74]. Reduced vascular tortuosity was also noted in MMD patients with *RNF213* Arg4810Lys compared with unaffected controls [54,75].

### 2.2. Japanese Studies on Arg4810Lys and MMD

Japanese studies have confirmed the association between Arg4810Lys and MMD consistently [76,77,78,79]. Ishigami et al. (2022) linked heterozygous carriers with bilateral symptoms and earlier MMD onset [80]. The mutation might increase the risk of progression from middle cerebral artery stenosis to MMD [81]. Hara et al. (2021) found Arg4810Lys to be associated with younger-onset MMD but absent in infantile cases, suggesting that its absence may be a biomarker for severe infantile MMD [82]. Familial cases demonstrated variable penetrance: one family showed a homozygous sibling with early-onset, rapidly progressing MMD and a heterozygous sibling with milder, later-onset disease [77].

Several individual cases with Arg4810Lys were published among Japanese patients [83,84,85,86,87,88,89,90,91,92,93,94,95]. The majority of them were patients with child onset. The affected patients were diagnosed with unilateral MMD, where carriers may have motor and speech difficulties. Among pregnant patients, the mutation may have an impact on hypertensive disorders. The mutation was also detected in asymptomatic relatives [84,88,89,90,91,92,93,94,95]. Interestingly, the Arg4810Lys wild-type genotype (GG) was identified as a risk factor for de novo hemorrhage in non-symptomatic MMD hemispheres [87]. Moteki et al. (2015) emphasized that besides Arg4810Lys, the significance of other rare *RNF213* risk variants, such as Thr3316Ile and Arg4062Gln, should not be overlooked [84]. Regional frequency differences in Japan should be considered with further confirmation [85,86].

### 2.3. Chinese and Other Asian Populations Studies on Arg4810Lys and MMD

In Chinese patients, Arg4810Lys is the most common variant among *RNF213* mutations in MMD and has been linked with abnormal collateral vessel formation and poor prognosis [43,44,46,96,97,98,99,100,101,102,103,104,105,106]. However, its frequency is lower than in other East Asian populations. Taiwanese studies reported a lower prevalence of Arg4810Lys than in mainland China, with incomplete penetrance [96]. In India, the variant was rare [107]. 

### 2.4. Biomarkers and Treatment Strategies for MMD

Biomarkers for MMD have been suggested as follows: Neuregulin 1 (NRG1) levels in serum are higher in Arg4810Lys carriers [108,109]. Thyroid autoantibodies appeared more often in MMD patients without the Arg4810Lys mutation [110]. Even though surgery remains essential, carriers undergoing bypass procedures (STA-MCA anastomosis or EDMAPS) present improved cerebral blood flow and reduced stroke recurrence risk [111,112,113,114,115,116,117,118]. Direct bypass surgeries can restore the blood flow faster than indirect surgeries, especially in homozygous carriers. Arg4810Lys has also been associated with an increased risk of postoperative temporal muscle swelling and delayed cerebral hyperperfusion [119,120]. Moreover, heterozygous Arg4810Lys carriers tend to develop better postoperative collateral formation after surgery [121]. Table 1 summarizes the differences among MMD patients with homozygous and heterozygous *RNF213* Arg4810Lys mutations.

## 3. Intracranial Artery Stenosis (ICAS) and Vascular Forms of Disease with *RNF213* Arg4810Lys

**Figure 2:**
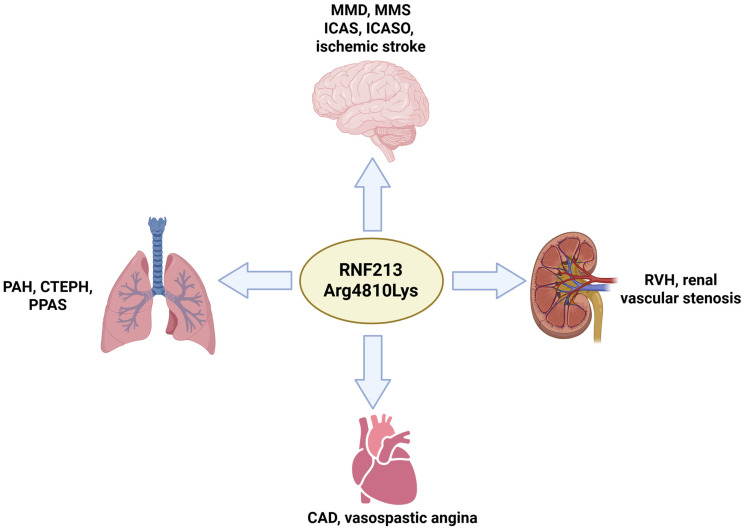
Summary of Arg4810Lys impact on diseases. The mutation can cause several conditions, associated with stenosis in the vessels of the brain (e.g., MMD, MMS, or ICAS), lung (PAH, PPAS, or CTEPH), heart (CAD or vasospastic angina), and kidney (RVH or renal vascular stenosis).

Besides MMD, the *RNF213* Arg4810Lys variant can impact multiple vascular dysfunctions, including intracranial artery stenosis (ICAS), intracranial artery stenosis/occlusion disease (ICASO), quasi-Moyamoya syndrome (MMS), and adult-onset ischemic stroke. Furthermore, *RNF213* Arg4810Lys affects the vascular system in other organs, such as the heart, lungs, and kidneys [50] (Figure 2).

ICAS is a major risk factor for ischemic or recurrent stroke, a common condition worldwide. *RNF213* Arg4810Lys has been suggested as a risk factor for ICAS [50,122,123,124,125,126]. In 2020, a Japanese study screened *RNF213* gene in 168 ICAS patients and 1194 controls, identifying 138 rare variants. They found a significant association between Arg4810Lys and ICAS, with a notable difference in variant frequency between patients and controls. However, the significance of other rare variants, such as Cys118Arg, Leu2356Phe, Ser193Gly, and Val1817Leu, suggested further study [126]. Genome-wide association studies (GWASs), combined with phenome-wide association studies, also confirmed the link between Arg4810Lys and ICAS. Associations were found between this variant and other risk factors, including hypertension and angina in ICAS patients [10]. Differentiating MMD from ICAS remains challenging, and additional future studies should be carried out to clarify the diagnosis and guide therapy [127].

*RNF213* gene has also been implicated in intracranial atherosclerosis. Kim et al. (2020) [121] reported that tandem lesions were more common in Arg4810Lys carriers than in non-carriers. This variant was independently associated with tandem sclerotic lesions regardless of other risk factors, such as hypertension, age, or smoking. Carriers also had a higher risk of stroke recurrence. These findings suggested that Arg4810Lys may modify the disease course and vascular pathology in East Asian patients [121,128,129,130]. Another Japanese study found that Arg4810Lys was associated with anterior ICAS but not with posterior ICAS [131]. A meta-analysis revealed that the variant increased the risk of ischemic stroke among patients with large-artery atherosclerosis [132]. *RNF213* Arg4810Lys was further associated with extracranial arterial stenosis, elevated maximum intima–media thickness in arteries, and increased stroke risk [133]. A notable Japanese case involved a 63-year-old man with ICAS after anastomosis surgery who developed de novo aneurysms in several vessels, including the temporal artery, middle cerebral artery, and external carotid artery, with radial artery–middle cerebral artery connections. It is possible that *RNF213* Arg4810Lys, alongside environmental factors, contributed to aneurysm formation [134].

Arg4810Lys has been strongly associated with intracranial artery stenosis/occlusion disease (ICASO) in Korean and Japanese patients, but this association is weaker in the Chinese or Indian population [135,136,137,138,139,140,141,142,143,144,145,146,147,148,149,150,151,152,153,154,155,156,157,158,159]. A Chinese study reported that Ala5021Val and Arg4810Lys may be risk factors for ischemic stroke related to ICASO, especially in women and younger-onset cases [135]. Although the variant was confirmed as a risk factor in East Asian populations, it occurred less frequently in Chinese ICASO patients in comparison with Korean and Japanese populations [136,137,138,139,140]. Matsuda et al. (2017) studied relatives of MMD patients and found that heterozygous Arg4810Lys carriers had intracranial stenotic lesions, unlike non-carriers, suggesting that this variant may be a biomarker for ICAS or ICASO risk, particularly in relatives of MMD patients [141]. Kamimura et al. (2019) reported that Arg4810Lys carriers with ICAS had an earlier onset of ischemic stroke compared with non-carriers [141]. Okazaki et al. (2022) [144] found that Arg4810Lys was a strong risk factor for intracranial artery stenosis, symptomatic stroke, and TIA, though it may not cause symptomatic stroke alone. Patients with this variant might progress to MMD in the future [144]. An analysis of patients with intracranial cervicocerebral artery dissections (IC-CADs) showed that *RNF213* Arg4810Lys was more common in patients than controls and was independently associated with IC-CAD apart from hypertension [145]. Furthermore, Arg4810Lys was a risk factor for intracranial arterial dissection of the middle cerebral artery [146].

An association exists between quasi-Moyamoya syndrome (quasi-MMD) or Moyamoya syndrome (MMS), characterized by comorbidities alongside classic MMD pathology and *RNF213* Arg4810Lys. The variant’s prevalence is lower in quasi-MMD than classic MMD, with the heterozygous variant being more common in arteriosclerotic or autoimmune quasi-MMD cases [147,148]. Cardiogenic cerebral embolism and Moyamoya-like with aplastic or twig-like middle cerebral artery (Ap/T-MCA) have also been reported [149].

An Indian study found a strong correlation between heterozygous Arg4810Lys and adult-onset ischemic stroke, with earlier onset and a positive family history [150]. A Chinese study reported a 0.5% heterozygous carrier frequency among acute ischemic stroke or TIA patients. Mutation carriers displayed younger onset and greater peripheral vascular disease prevalence. Arg4810Lys was associated with large-artery atherosclerosis, anterior circulation stenosis, and extracranial arterial stenosis but did not predict poorer prognosis or recurrence [153,154]. Eto et al. (2022) found a strong association between Arg4810Lys and circle of Willis abnormalities in cerebrovascular disease patients; carriers tended to form posterior communicating arteries more but anterior communicating arteries less frequently than non-carriers [155].

An additional atypical case was reported, a case of *RNF213*-related vasculopathy presenting with hemichorea, which suggested the impairment of angiogenesis and basal ganglia circuitry, leading to chorea-like symptoms [156]. Two patients with familial partial lipodystrophy and MMD-like vascular lesions carried heterozygous Arg4810Lys but no mutation in known lipodystrophy genes. Both had diabetes, abnormal fat distribution, and cerebrovascular events, suggesting that Arg4810Lys might be a risk modifier in lipodystrophy-related vascular disease [159]. Table 2 summarizes the brain diseases and clinical phenotypes of *RNF213* Arg4810Lys carriers.

## 4. Non-Neurological Forms of *RNF213* Arg4810Lys

### 4.1. RNF213 Arg4810Lys and Pulmonary Dysfunctions

The *RNF213* Arg4810Lys mutation can exhibit significant impact on both cerebral and pulmonary vasculatures. Several cases have been reported in patients with *RNF213* Arg4810Lys and pulmonary vascular diseases, such as pulmonary arterial hypertension (PAH) or peripheral pulmonary arterial stenosis (PPAS). While the precise disease mechanisms remain under investigation, critical observations suggest that Arg4810Lys may have a role in severe and often treatment-resistant pulmonary vascular diseases [160]. This mutation is linked with poor clinical outcomes in patients with idiopathic PAH, even after conventional therapies, suggesting that carriers may benefit from early lung transplantation due to the aggressive nature of the disease presentation [1,160,161,162,163,164,165,166,167,168,169,170]. The *RNF213* Arg4810Lys mutation demonstrates varying phenotypes based on its zygosity. Homozygous carriers frequently present with severe manifestations, including adult-onset PPAS with characteristic diffuse stenosis and MMD or MMD-like intracranial stenosis. This severe presentation reveals the comprehensive impairment of both cerebrovascular and pulmonary vascular systems in homozygous individuals. In contrast, heterozygous carriers, while also susceptible to idiopathic PAH [1,161,166], may exhibit different disease courses, and their role in pediatric peripheral pulmonary arterial stenosis (PPAS) [164,165,166,170] and chronic thromboembolic pulmonary hypertension (CTEPH) may not be ruled out [169] without overt stenosis warrants further investigation to clarify the full spectrum of the mutation’s influence. Inflammation was reported as a recurrent pathological feature observed in patients with the *RNF213* Arg4810Lys mutation and pulmonary hypertension [1,160,161,162,163,164,165,166,167,168,169,170].

### 4.2. RNF213 Arg4810Lys and Coronary Artery Disease (CAD) and Kidney Dysfunctions

*RNF213* Arg4810Lys has also been linked with coronary artery disease (CAD) in Japanese patients [171]. A familial case Arg4810Lys with CAD was reported, where other family members were diagnosed with typical MMD [36,172,173,174,175,176]. Furthermore, *RNF213* Arg4810Lys was implicated in CAD in a Japanese population, with a higher allele frequency in CAD patients than in the controls [172]. Koizumi et al. (2012) found an association between Arg4810Lys and systolic blood pressure [173]. Genome-wide association studies revealed that Arg4810Lys correlated with vasospastic angina (VSA) in male patients, especially young-onset cases. Homozygous Arg4810Lys is a stronger risk factor than heterozygous forms and has also been linked with increased mortality in VSA patients without CAD, likely due to coronary vasomotor dysfunction rather than atherosclerosis [174,175,176].

In rare cases, MMD patients with *RNF213* Arg4810Lys develop renal artery stenosis (RVS), although it is unclear whether the variant directly impacts the kidney vasculature [177]. Another study suggested a role for Arg4810Lys in renovascular hypertension (RVH) in children [178].

## 5. *RNF213* Arg418Lys and Modifier Factors

There have been several reported cases in which *RNF213* Arg4810Lys coexisted with other variants, leading to vascular impairment or increasing the severity of patients’ conditions [179,180,181,182,183,184,185,186,187,188,189,190,191,192,193,194,195,196] (Supplementary Table S1). Two compound heterozygous cases where *RNF213* Arg4810Lys coexisted with the TNF213 Thr1727Met mutation were found. In both cases, MMD phenotypes were present, but atypical symptoms, such as systemic lupus erythematosus (SLE), were also reported [180,181]. Another compound heterozygous case of the *RNF213* Arg4810Lys mutation was observed along with the Ser3986Asn mutation in a patient with Graves’ disease 182,183]. *RNF213* Arg4810Lys also coexists with other pathogenic mutations or chromosomal abnormalities, including trisomy, *NOTCH3*, *DMD*, *FLNA*, or *CBL* mutations [184,185,186,187,188,189,190,191,192,193,194,195,196].

### 5.1. Compound Heterozygous Cases

A 46-year-old patient developed atherosclerotic moyamoya disease (MMD) and was compound with heterozygous for *RNF213* Arg4810Lys and Thr1727Met. Her initial symptom was slurred speech. Stenosis was detected in several vascular regions, including the middle cerebral artery (MCA), bilateral anterior cerebral artery (ACA), and left posterior cerebral artery (PCA). Moyamoya vessels were also present in her brain. Despite receiving antiplatelet and statin therapy, MCA stenosis on the left side progressed, and the number of moyamoya vessels increased [180]. Another case involved a 32-year-old female patient carrying both the Arg4810Lys and Thr1727Met mutations who presented with cerebral infarction due to MMD and systemic lupus erythematosus (SLE). She exhibited weakness in her left limb and visual impairment; immunoglobulin levels were also abnormal [181]. *RNF213* Arg4810Lys was also reported alongside Ser3986Asn in a diagnosed Japanese female with MMD concurrent with Graves’ disease (GD). At age 29, she developed progressive cerebral infarctions in bilateral hemispheres during thyrotoxicosis. She underwent revascularization surgery and thyroid hormone therapy, after which her condition improved [182,183].

### 5.2. RNF213 Arg4810Lys and Other Gene Mutations

In several cases, *RNF213* Arh4810Lys coexists with other disease-related gene mutations. These patients present both vascular abnormalities and other disease phenotypes [184,185,186,187,188,189,190,191,192,193,194,195,196]. A study emphasized the phenotypic diversity, despite shared mutations, and suggested that environmental factors influence expression. The *RNF213* Arg4810Lys variant has been verified to be a main risk factor for MMD, but it could also be a significant disease modifier in the case of other complex genetic disorders, leading to diverse clinical presentations [184,185,186,187,188,189,190,191,192,193,194,195,196]. The coexistence of MMD with Down syndrome (DS) was reported. These children developed neurological deficits due to fresh infarction, stroke, or cerebral hemorrhage [184,185]. *RNF213* Arg4810Lys may also act as a risk modifier in Duchenne muscular dystrophy (DMD). A family in which Arg4810Lys coexisted with c.9953_9954delAG in the *DMD* gene was identified. The female carriers of both variants presented vascular dysfunctions, such as episodic rhabdomyolysis and cardiac abnormalities transient ischemic attacks, numbness, weakness, or ICA stenosis without motor issues. Their brother showed typical DMD symptoms and died of cardiopulmonary problems [186]. *RNF213* Arg4810Lys and KIF1A gene Ala85Asp coexistence was found in an atypical MMD case with gait disturbance, childhood epilepsy and intellectual disability, spastic paraplegia, and cerebellar atrophy [187]. *RNF213* Arg4810Lys and a splice site variant in the Noonan syndrome-associated CBL gene (c.1228-2 A>G) were found in a case of left main coronary artery ostial atresia (LMCAOA) [188]. *RNF213* R4810K coexisted with filamin A (*FLNA*) Gly1623Val fs*41 in a patient exhibiting Ehlers–Danlos-like symptoms and bilateral periventricular nodular heterotopia (PNH), but seizures and MMD-like cerebral vascular formation also appeared [189]. A Korean *RNF213* Arg4810Lys and an *AFF4* mutation (Pro253Leu) was reported along with CHOPS syndrome and MMD, developmental delay, short stature, synophrys, intellectual disability, and vascular abnormalities in renal arteries and the infrarenal aorta [190]. *RNF213* Arg4810Lys coexisted with *PCSK9* Glu32Lys (familial hypercholesterolemia) in a Japanese patient with multiple intracranial major artery stenoses and asymptomatic intracranial atherosclerotic stenosis (ICAS). PCSK9 and RNF213 proteins may interact via lipid metabolic pathways [191]. Another Japanese study confirmed *RNF213* Arg4810Lys’s involvement with familial hypercholesterolemia genes, which may cause more severe stenosis or occlusion in the anterior circulation and increase the risk of intracranial major artery stenosis/occlusion (ICASO) [192]. *RNF213* Arg4810Lys was examined in CADASIL patients with NOTCH3 cysteine-affecting mutations; carriers had a higher intracranial artery stenosis (ICAS) risk [193]. A 66-year-old patient with three strokes starting at age 62 carried both the *RNF213* variant and a *NOTCH3* mutation (Cys1250Arg). Imaging showed infarcts in the left temporal pole and middle cerebral artery stenosis. He was diagnosed with CADASIL. The variant may modify vascular risk in CADASIL [193]. *RNF213* Arg4810Lys may also contribute to quasi-MMD onset in neurofibromatosis type 1 (NF1) patients [152]. A Chinese study investigated MMD patients carrying the *RNF213* Arg4810Lys mutation and a variant in hyaluronan- and proteoglycan-binding link protein 3 (*HAPLN3*) Thr34Ala. The *HAPLN3* variant appeared to act as a modifier in Arg4810Lys carriers by increasing tube formation and VEGF expression in endothelial cells, thereby contributing to the incomplete penetrance of MMD [194]. A Japanese study found that the HLA allele HLA-DRB1*04:10 impacted the thyroid disease in MMD patients but found no association between *RNF213* Arg4810Lys and this allele, suggesting that Arg4810Lys may not directly influence antigen recognition or presentation mechanisms [195]. A Chinese study investigated interactions between *RNF213* variants and other genes, including *PDGFRB*, *MMP-3*, and *TIMP-2*. While confirming *RNF213* Arg4810Lys as a risk factor for MMD, no interaction was found with these genes, indicating that larger population studies are needed to explore possible genetic interactions [179].

### 5.3. RNF213 Arg4810Lys and Environmental Modifiers

Environmental factors, including hypoxia, inflammatory factors, and lifestyle factors, have been reported to impact strongly the clinical phenotypes of patients with the Arg4810Lys mutation [196]. Inflammatory factors have been suggested to play a crucial role in the regulation of *RNF213* expression and angiogenesis [196]. Hypoxia was found to be one of the environmental factors which may impact the disease course in Arg4810Lys carriers. Animal models revealed that cerebral hypoxia may impair angiogenesis, leading to vascular dysfunction or more severe disease phenotypes. Hypoxia may affect both MMD and PAH, leading to disease onset in asymptomatic carriers, or it could result in more severe disease phenotypes [161,196].

Furthermore, lifestyle factors, infections, or certain surgeries have been found to impact the clinical phenotypes of *RNF213* Arg4810Lys. Hypertension has been verified to be a strong risk factor for ICAS development in case of *RNF213* mutations, including Arg4810Lys [123,176]. Dyslipidemia has been found to be a risk factor for ICAS onset, since abnormal lipid levels are correlated with ICAS prevalence [197]. Furthermore, smoking or alcohol consumption can be risk factors for vascular dysfunctions. It cannot be ruled out that these factors could result in more severe phenotypes of MMD or ICAS in the case of *RNF213* Arg4810Lys carriers [123]. Physical activity can reduce the risk for vascular dysfunctions. It would be worth investigating whether regular physical activity can prevent vascular impairment in *RNF213* Arg4810Lys carriers or reduce the severity of symptoms in patients [24].

Microbial infections may also impact the clinical outcomes of patients with *RNF213* Arg4810Lys. As mentioned before, RNF213 can be involved in protection against different pathogens. A reversible cerebral angiopathy case associated with *RNF213* Arg4810Lys was reported after a patient contracted hand, foot, and mouth disease. Follow-up imaging showed resolution of initial bilateral carotid stenosis and infarction, but further studies should be carried out on viral effects in mutation carriers [158]. Another case of an *RNF213* Arg4810Lys carrier developed Herpes simplex virus-1-related infantile encephalitis but also developed stroke and vascular abnormalities. It could not be ruled out that HSV-1 infection had some impact on the early development of vascular symptoms in the presence of the mutation [198]. It cannot be ruled out that microbial infections could be trigger mechanisms in individuals with the mutation.

Medical procedures, such as radiation therapy, were found to impact clinical outcomes in *RNF213* Arg4810Lys carriers. Radiation may also impact the MMS phenotype in case of Arg4810Lys. A child with a brain tumor received proton beam therapy and developed vascular issues, such as stenosis in the circle of Willis arteries and the ICA, with repeated transient hemiparesis and ischemic stroke episodes. Revascularization surgery improved his condition. However, further studies are needed on how radiation could impact vascular phenotypes in the case of the Arg4810Lsy mutation [154]. Brain arteriovenous malformation (AVM) in case of de novo MMD also appeared after stereotactic radiosurgery (SRS), suggesting AVM may increase MMD risk in the presence of *RNF213* variants like Arg4810Lys [157].

## 6. Functional Studies and Pathogenic Pathways of *RNF213* Arg4810Lys

The *RNF213* Arg4810Lys mutation, a primary risk factor for MMD, compromises vascular integrity and function through a convergence of pathological mechanisms. While initial computational analyses of this mutation yielded conflicting results, further research has suggested that it may alter RNF213′s intrinsic enzymatic activity and its interaction with critical cellular pathways. For example, the mutation has been hypothesized to increase iron (or heme) binding affinity and affect insulin signaling by altering insulin receptor binding. This mechanistic change could lead to reduced phosphorylation of the AAA+ ATPase domain, consequently decreasing enzyme activity and potentially contributing to insulin resistance in MMD patients [45]. Extensive cellular and animal model studies have been conducted to understand how the *RNF213* Arg4810Lys mutation impairs vascular development and repair, disrupts cell proliferation, and exacerbates cellular stress responses, thus providing a unifying framework for its diverse pathological manifestations [199,200,201,202,203,204,205,206,207,208,209,210,211,212,213].

The *RNF213* Arg4810Lys variant consistently impacts angiogenesis, leading to vasculopathy characterized by the negative remodeling of both intracranial and systemic blood vessels [57,199,200,201,202,203,204,205,206]. Morito et al. [203] suggested that induced pluripotent stem cells (iPSCs) from MMD patients harboring Arg4810Lys exhibited impaired angiogenesis. Extracellular matrix (ECM) receptor-related genes were downregulated in these cells, while proteomic analysis showed lower levels of cytoskeletal proteins but upregulation of splicing-related proteins. This suggests that the downregulation of ECM receptor genes and the upregulation of splicing genes may negatively impact angiogenesis [203]. Cell studies on iPSCs derived from patients homozygous or heterozygous for Arg4810Lys revealed reduced angiogenic activity, as indicated by lower expression of markers such as CD34(+), CD133(+), and KDR(+), in comparison with controls. Circulating endothelial progenitor cells (EPCs) were also reduced in children with MMD, leading to abnormal vascular differentiation, impaired tube formation, and reduced vascular repair [202]. Another study confirmed that *RNF213* Arg4810Lys did not prevent arteriogenesis but inhibited angiogenesis and reduced cerebral blood flow [204]. *RNF213* Arg4810Lys has been suggested to be a loss-of-function mutation, though some gain-of-function effects may also exist. Zebrafish models carrying Arg4810Lys showed abnormal vessel formation, including irregular blood vessel walls and impaired sprouting. However, the mutation did not affect RNF213 transcription or ubiquitination levels [56,207,208]. Fibroblasts from MMD patients with Arg4810Lys and other mutations expressed higher levels of matrix metalloprotease 1 (MMP1) mRNA than controls. Normal RNF213 may play a role in downregulating MMP expression in endothelial cells, facilitating proper angiogenesis [208]. Notably, Arg4810Lys has been suggested not to impair lipid metabolism [209]. The mutation leads to the upregulation of inflammatory signals, including interferons (IFNs), in various cell lines, such as iPSCs or human umbilical vein endothelial cells (HUVECs). Increased IFN levels may reduce angiogenesis. Mouse models expressing Arg4810Lys exhibited inhibited angiogenesis under hypoxia and interferon exposure. This variant may reduce RNF213 ATPase activity, which also contributes to impaired angiogenesis [196]. Liu et al. (2011) reported that Arg4810Lys did not alter RNF213 expression levels [56]. However, transfection of the variant into iPSCs was associated with reduced angiogenic activity [201,204,205].

The overexpression of Arg4810Lys in cell lines also reduced the expression of mitosis-related genes, including securin, leading to decreased angiogenesis [201,204,205]. HeLa cells carrying *RNF213* Arg4810Lys formed fewer colonies during mitosis and exhibited significantly longer mitotic duration compared with wild-type cells. The overexpression of the mutation caused accumulation of cells in the G2/M phase with higher DNA content. Approximately 15% of mutant cells failed cytokinesis, resulting in incomplete cell division. These cells showed increased complex formation with mitotic arrest deficiency 2 (MAD2), causing MAD2 mislocalization and inactivation. Since MAD2 depletion impaired mitosis and downregulated securin expression, iPSC lines from MMD patients revealed similar mitotic failures, including prolonged prometaphase-to-metaphase transition. The study indicated that Arg4810Lys increases the risk of aneuploidy [206].

Comparisons between MMD patient-derived peripheral blood mononuclear cells (PBMCs) and controls, along with HUVECs transfected with Arg4810Lys or wild-type RNF213, suggested that the variant impaired the autophagy-related pathways following oxygen–glucose deprivation (OGD). Environmental stress inhibited autophagy by stimulating SQSTM1/p62 and LC3 expression, causing autophagosome accumulation and defective clearance, which may contribute to vascular impairment and MMD progression [210]. The mutation was also found to reduce global ubiquitination. HeLa cells expressing Arg4810Lys exhibited decreased auto-ubiquitination activity compared with those expressing wild-type RNF213 [211].

**Figure 3:**
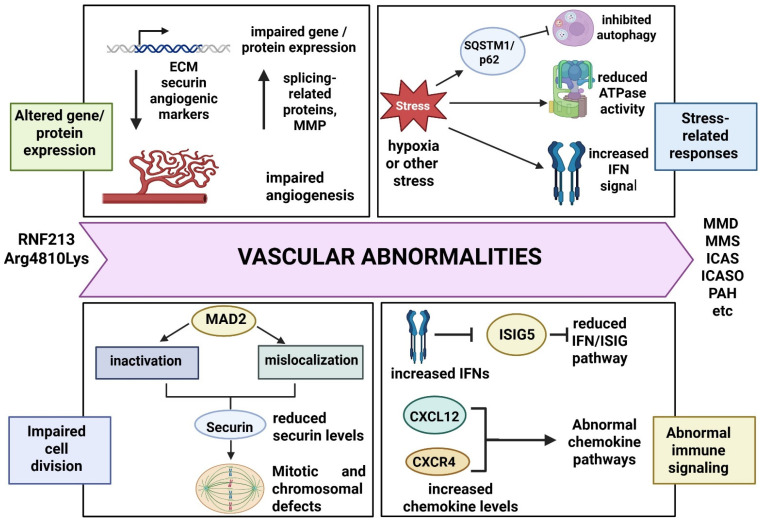
Potential disease-related mechanisms of RNF213 Arg4810Lys. The mutation may impact diseases through different mechanisms, including altered expression of angiogenesis-related genes, leading to impaired angiogenesis. Hypoxia and stress may also induce pathogenic mechanisms, including inhibited autophagy, lower ATPase activity and higher pro-inflammatory expression. Arg4810Lys could also impair cell division through reduced securin expression. Furthermore, Arg4810Lys could result in impaired immune signaling.

*RNF213* Arg4810Lys has been associated with impaired T-cell activation and proliferation and may cause abnormal antigen presentation, leading to aberrant T-cell responses [207]. Mouse models expressing Arg4810Lys showed normal development of intracranial arteries and the circle of Willis, suggesting that mutant mice do not spontaneously develop MMD. However, their vascular system may be more sensitive to environmental factors [208]. As mentioned, *RNF213* Arg4810Lys may be involved in pulmonary arterial hypertension (PAH). Mouse models with this mutation demonstrated increased ventricular systolic pressure and a thickening of pulmonary medial arterial walls after hypoxic exposure. The mutation may impair non-canonical Wnt signaling, since the levels of the chemokines C-X-C motif chemokine ligand 12 (CXCL12) and its receptor, C-X-C chemokine receptor type 4 (CXCR4), were elevated in mutant mice. The study confirmed that the Arg4810Lys variant impairs chemokine-related pathways, including the CXCL12/CXCR4 signaling axis [213]. Figure 3 summarizes the potential disease-related mechanisms of *RNF213* Arg4810Lys based on cell and animal studies, including altered gene and protein expression, abnormal immune responses, impaired angiogenesis, reduced ATPase activity, or inhibited autophagy.

## 7. Discussion

Strong associations exist between the *RNF213* Arg4810Lys variant and various vascular diseases, including MMD. This variant is remarkably prevalent in East Asian populations, with reported frequencies ranging from 1% to as high as 2.5% among people in Japan, Korea, and China. The elevated frequency of Arg4810Lys in East Asians is attributed to the founder effect [68,80,150]. Although this mutation has also been detected in Indian patients with ischemic stroke, its frequency in India appears to be lower than in East Asia, warranting further studies on its prevalence within Indian populations [150]. In contrast, *RNF213* Arg4810Lys is rare or unreported in other populations, including Caucasian and South Asian groups [68].

The *RNF213* Arg4810Lys mutation significantly contributes to a range of vasculopathies, including intracranial artery stenosis, extracranial stenosis, and pulmonary hypertension, as well as coronary and renal artery diseases [50,90,123]. Carriers of Arg4810Lys are more likely to develop MMD with transient ischemic attacks (TIAs) or strokes, although the age of onset among carriers varies widely [50]. Additionally, this variant has been linked with abnormalities in the pulmonary vascular system, including pulmonary arterial hypertension (PAH). Disease prognosis is often poorer in carriers than in non-carriers [161,162]. *RNF213* Arg4810Lys is increasingly recognized for its implications in coronary artery disease (CAD) and related cardiovascular conditions [174,175,176]. It has also been associated with renal artery stenosis, leading to renovascular hypertension [177,178]. Collectively, the presence of this variant can trigger systemic vasculopathy, causing vascular abnormalities that impair the blood flow and arterial integrity across multiple organ systems (Table 3) [50].

Several studies have investigated the functional role of *RNF213* Arg4810Lys in the vascular system. The mutation appears to disrupt pathways essential to vascular development and endothelial function, resulting in abnormal angiogenesis and blood vessel formation. It also significantly impacts autophagy, leading to accumulation of proteins such as SQSTM1/p62, a marker of impaired autophagic degradation [123,199,200,201,202,203,204,205,206,207,208,209,210,211,212,213]. Moreover, the mutation influences the immunological pathways, including elevated production of pro-inflammatory signals such as interferons [208]. Animal models further suggest that Arg4810Lys affects genomic stability and may cause mitotic abnormalities [206].

The gene dosage of *RNF213* Arg4810Lys has been suggested to play a significant role in clinical outcomes. Both homozygous and heterozygous carriers exhibit vascular disease phenotypes; however, homozygous individuals typically experience earlier onset and more severe disease. Conversely, heterozygous carriers may remain asymptomatic throughout their lives due to the mutation’s incomplete penetrance, which is influenced by other genetic, environmental, and lifestyle factors [50,78,166,198,214,215,216,217,218,219,220,221]. Animal studies have shown that environmental stressors, such as hypoxia, could trigger pathogenic features, including reduced ATPase activity and increased inflammatory signaling in Arg4810Lys mutant mice, which are otherwise absent under normal conditions, accentuating the importance of external factors in disease manifestation [209].

Vascular health is another critical factor shaping disease outcomes in Arg4810Lys carriers. Hypertension can exacerbate vascular remodeling associated with MMD in these individuals [222]. Environmental factors, such as air pollution and toxin exposure, may also contribute by increasing oxidative stress, which, together with the mutation, impairs the endothelial function and promotes disease progression [161,222].

In several reported cases, *RNF213* Arg4810Lys coexisted with other mutations, including trisomy, *KIF1A*, *FLNA*, *PCSK9*, and *NOTCH3*. In most instances, Arg4810Lys worsened the patients’ conditions, suggesting that it may share pathogenic pathways with other genetic risk factors. For example, Down syndrome patients carrying this mutation face higher risks of vascular dysfunction and complications, indicating that RNF213 could act as a disease modifier in these contexts [179,180,181,182,183,184,185,186,187,188,189,190,191,192,193,194,195,196]. Coexisting mutations could have additive or synergistic effects on disease expression: *FLNA* variants could promote vascular abnormalities alongside Arg4810Lys; *NOTCH3* mutations combined with Arg4810Lys lead to more severe cerebrovascular disease; and *PCSK9* variants influence the cardiovascular phenotypes that modify Arg4810Lys-related vascular risk. Overall, such genetic interactions may modulate the penetrance, severity, and clinical heterogeneity of Arg4810Lys-associated vasculopathies [179,180,181,182,183,184,185,186,187,188,189,190,191,192,193,194,195,196,223,224].

## 8. Conclusions

As a common variant among East Asian patients, *RNF213* Arg4810Lys serves as a useful marker distinguishing MMD patients from healthy controls. Its strong association with MMD supports its use for early and accurate diagnosis, especially in carriers presenting with vascular abnormalities, such as unilateral or bilateral intracranial artery stenosis, or occlusion lesions suggestive of MMD [55,68,80]. Moreover, the presence of Arg4810Lys in MMD patients correlates well with poorer prognosis, including a higher frequency of ischemic symptoms and an increased risk of progressive cerebrovascular stenosis compared with non-carriers. Identifying this variant would improve risk profiling and assist in determining the need and timing for revascularization surgery. Genetic testing could also aid in anticipating postoperative collateral formation and functional outcomes, and incorporating *RNF213* Arg4810Lys status into clinical practice could enhance diagnosis, prognosis prediction, and surgical management in MMD patients [80,123,225,226]. Genetic counseling for carriers and their families should include a comprehensive evaluation of recurrence risk, education about the mutation’s variable penetrance and expressivity, and discussion of possible clinical manifestations, such as MMD, ischemic stroke, pulmonary arterial diseases, and other systemic vasculopathies [227,228,229].

## Figures and Tables

**Table 1:** MMD patients with Arg4810Lys in homozygous vs. heterozygous form.

	Heterozygous Arg4810Lys	Homozygous Arg4810Lys
Penetrance	Lower	Higher
Vascular features	Less severe	More extensive stenosis
Stroke outcome	Increased risk of ischemic stroke; prognosis is less severe	Higher risk of recurrent strokes; prognosis is worse
Age at onset	Later onset	Earlier onset
Disease progression	Relatively slow	Faster disease progression
Treatment response	Good response to revascularization surgery or other treatment	Needs early and aggressive management; outcome may be poor

**Table 2:** *RNF213*-related vascular diseases in the brain of patients with *RNF213* Arg4810Lys.

Disease	Zygosity	Clinical Phenotypes	Prognosis
MMD	Heterozygous/homozygous	TIAs, stroke, hemorrhage, seizures, and cognitive decline	Revascularization surgery may be successful
ICAS/ICASO	Heterozygous	Stroke, tandem lesions, and anterior circulation stenosis	Risk of progression to MMD
MMS	Heterozygous	Atypical MMD + comorbidities	Variable severity
Dissections	Heterozygous	Sudden stroke and MCA dissection	Early onset and treatable

**Table 3:** Summary of *RNF213* Arg4810Lys-related vascular diseases and phenotypes in other organs.

Organ	Disease	Key Symptoms/Risks	Prognostic Outcome
Lungs	PAH and PPAS	Dyspnea and poor therapy response	Lung transplant in severe cases
Heart	CAD and VSA	Angina and vasospasm	Higher mortality in males/homozygous cases
Kidney	RVA and RVS	Hypertension and ischemia	Rare but possible

## Supplementary Materials

The following supporting information can be downloaded at https://www.mdpi.com/article/10.3390/ijms26167864/s1 [180,181,182,183,184,185,186,187,188,189,190,191,192,193,195].
