# Supplementary material for: Multisystemic Impact of RNF213 Arg4810Lys: A Comprehensive Review of Moyamoya Disease and Associated Vasculopathies
Source: Int J Mol Sci. 2025 Aug 14;26(16):7864. doi: 10.3390/ijms26167864 (PMC12386203; doi:10.3390/ijms26167864)
Supplement: Supplementary file 1 [file ijms-26-07864-s001.zip › ijms-3774026-supplementary.pdf]

Supplementary Table S1. RNF213 Arg4810Lys co-existence with other variants.

Other mutation	Age	Disease	symptoms	Vascular impairment	Reference
RNF213 Thr1727Met	46 years	MMD	Slurred speech	stenosis in vascular areas	[162].
RNF213 Thr1727Met	32 years	MMD and SLE	Motor-and visual impairment	cerebral infarction	[163]
RNF213 Ser3986As	29 years	MMD, Graves disease	NA	NA	[164, 165].
KIF1A Ala85Asp	childhood	spastic paraplegia	epileptic fits and intellectual disability, motor impairment	no cerebral ischemia, cerebral vessel abnormalities	[169]
Trisomy	childhood	DS and MMD	Stroke at 9 years of age, vomiting headache at the age of 13	hemorrhage occurred in the anterior choroidal	[166]
Trisomy	2 years	DS and MMD	Motor impairment	fresh cerebral infarction	[167]
DMD c.9953_9954 delAG	Child onset	MMD/ DMD	episodic rhabdomyolysis or Muscle impairment	ICA stenosis can occur	[168]
CBL c.1228-2 A >	14 years	LMCAOA	Heart dysfunctions	coronary artery issues	[170]
FLNA Gly1623Val fs*41	21 years	Ehlers-Danlos-like symptoms	Seizures, headaches and motor impairments (joint hypermobility)	MMD-like vascular formation	[171]
AFF4 Pro253Leu	6 years	CHOPS syndrome, MMD	Developmental delay, intellectual disability	Narrowed renal arteries and aorta	[172]
PCSK9 Glu32Lys	75 years	ICAS	No specific symptoms	Stenosis in brain	[173, 174]
NOTCH3 Cys1250Arg	62 years	CADASIL	Stokes	ipsilateral middle cerebral artery stenosis in the, brain infracts	[176]
HLA-DRB1*04:10 allele	NA	Thyroid disease, MMD	NA	NA	[178]
